# Supplementary figures and images for: Autocrine feedback maintains homeostatic neuropeptide expression in a peptidergic hub neuron
Source: bioRxiv. 2026 Jan 10:2026.01.09.698615. Preprint. [Version 1] doi: 10.64898/2026.01.09.698615 (PMC12803089; doi:10.64898/2026.01.09.698615)

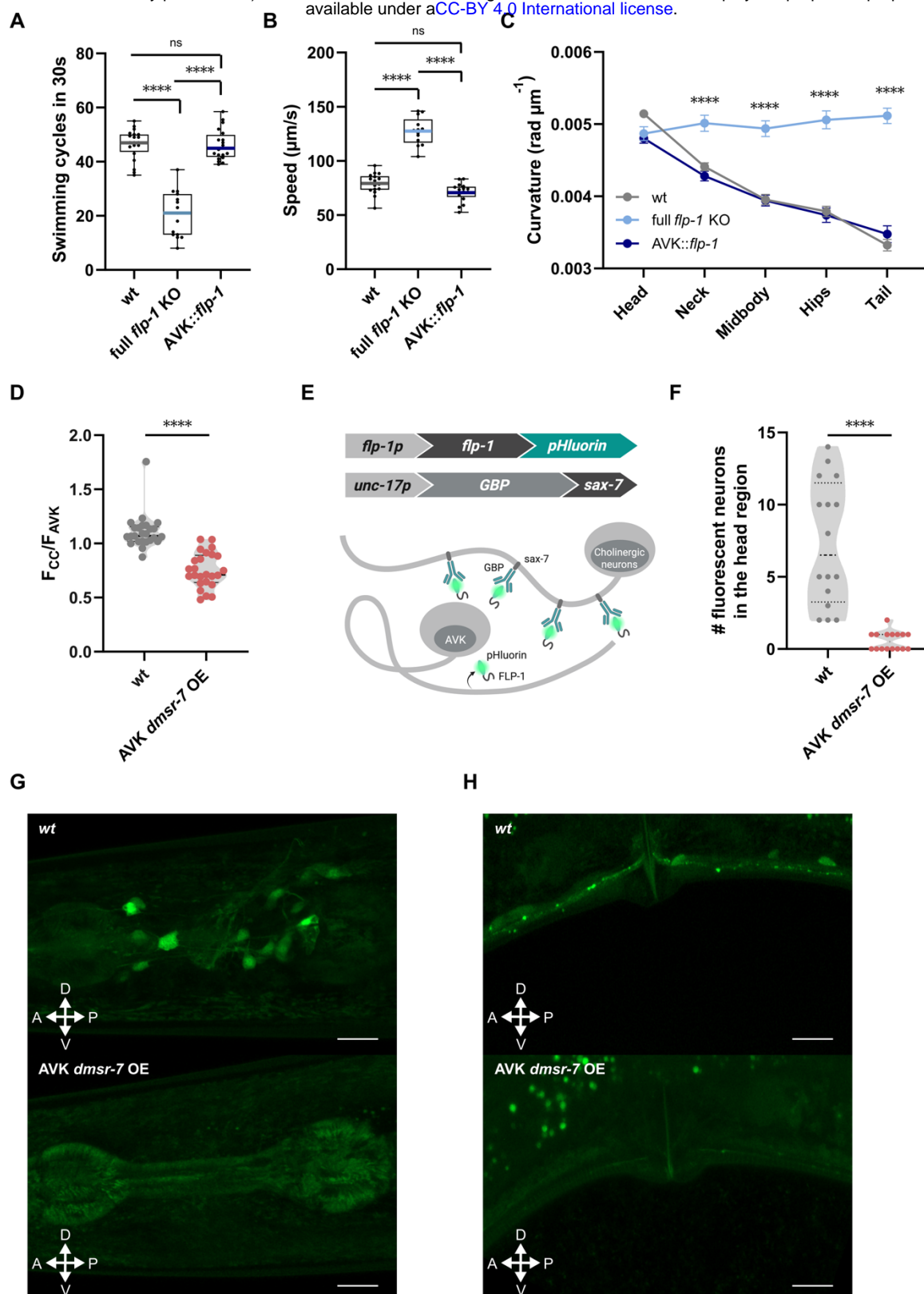

Figure S1.



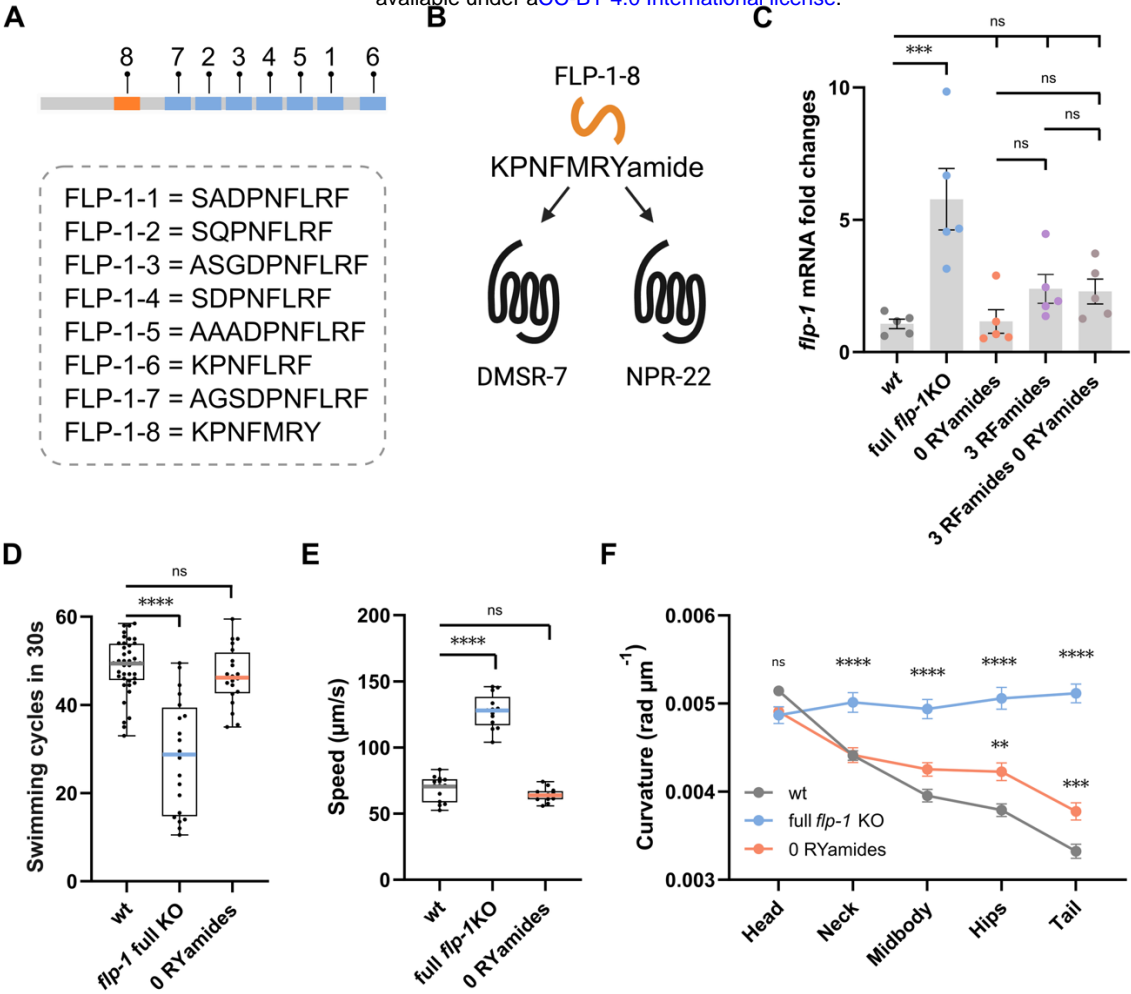

Figure S3.

Supplement: Supplement 1 — Figure S1. Quantification and visualization of FLP-1 distribution in wild-type and dmsr-7-overexpressing animals. (A) Swimming cycles of flp-1 mutant animals and mutants rescued in AVK. (B and C) Speed and curvature for full flp-1 mutants and rescued animals in AVK neurons. (D) Mean fluorescence intensity of FLP-1::mKate in coelomocytes, normalized to AVK fluorescence, quantified in wild-type and dmsr-7-overexpressing animals. (E) Schematic of the biochemical strategy used to visualize FLP-1 distribution. FLP-1 peptide, expressed under its promoter and tagged with pHluorin, interacts with membrane-anchored (sax-7) GFP Binding Protein (GBP) expressed in cholinergic neurons (under unc-17p). (F) Number of fluorescent neurons in the head of wild type and animals overexpressing dmsr-7 in AVK. (G and H) Confocal images of cholinergic neurons tethering released FLP-1::pHluorin in head (D) and vulva (E) regions of wild-type and dmsr-7-overexpressing animals. Scale bar = 15 μm. Data represent mean ± SEM from (A to C) ≥ 14 individual plates with 10 animals in each, and (D and F) ≥ 15 animals per genotype. Statistical analysis performed using one-way ANOVA with post hoc Dunnett’s tests (A), Tukey test in (B and C) and t-test for (D and F); ****P < 0.0001. Figure S2. Expression and regulation of neuropeptides in AVK neurons. (A) Z-score values of neuropeptides expressed in AVK neurons across the entire nervous system of L4-stage animals 34 . (B) Transcripts per million (TPM) expression values of neuropeptides detected in AVK neurons. (C) Relative fold change in neuropeptide expression in wild-type versus flp-1 knockout animals from whole-mount animal mRNA extrats. Data represent mean ± SEM from N = 3 independent assays. Statistical analysis was performed using one-way ANOVA followed by post hoc Tukey correction; ****P < 0.0001 ***P < 0.001, *P < 0.05. Figure S3. FLP-1 RYamide signaling doesn’t regulate locomotor behavior. (A) Sequences for individual FLP-1 peptides retrieved fr [file NIHPP2026.01.09.698615v1-supplement-1.pdf]
